# Hypoglycemic Effects of *Silphium perfoliatum* L. In Vitro and In Vivo and Its Active Composition Identification by UPLC-Triple-TOF-MS/MS

**DOI:** 10.3390/ph18081087

**Published:** 2025-07-23

**Authors:** Guoying Zhang, Liying Liu, Wenjing Jia, Luya Wang, Jihong Tao, Wei Zhang, Huilan Yue, Dejun Zhang, Xiaohui Zhao

**Affiliations:** 1 Research Centre for High Altitude Medicine, Key Laboratory of High Altitude Medicine (Ministry of Education), Key Laboratory of Applied Fundamentals of High Altitude Medicine (Qinghai-Utah Joint Research Lab for High Altitude Medicine), Laboratory for High Altitude Medicine of Qinghai Province, Qinghai University, Xining 810001, China; 2 Qinghai Provincial Center for Drug Evaluation and Inspection, Xining 810007, China; 3 Qinghai Key Laboratory of Qinghai-Tibetan Plateau Biological Resources, Qinghai Provincial Key Laboratory of Tibetan Medicine Research, Northwest Institute of Plateau Biology, CAS, Xining 810008, Chinahlyue@nwipb.cas.cn (H.Y.); 4 University of Chinese Academy of Sciences, Beijing 101408, China; 5 Qinghai Provincial Drug Inspection and Testing Institute, Xining 810016, China

**Keywords:** hypoglycemic effect, *Silphium perfoliatum* L., UPLC-triple-TOF-MS/MS, *α*-glucosidase inhibition, caffeoylquinic acids, flavonol glycosides

## Abstract

**Background:** Reducing postprandial blood glucose (PBG) is a crucial strategy for treating diabetes and minimizing the risk of complications. Developing efficient and safe *α*-glycosidase inhibitors from natural products to lower PBG has attracted much attention. *Silphium perfoliatum* L. (SP), a traditional herbal medicine of North American Indigenous tribes, has efficacy of treating metabolic diseases, but its hypoglycemic activity and bioactive components have not been fully studied. **Methods:** In vitro *α*-glucosidase inhibition and in vivo sucrose/maltose/starch tolerance assays were performed to assess the hypoglycemic effects of SP extracts, and UPLC-Triple-TOF-MS/MS analysis was used to tentatively identify its chemical structure composition. In vitro enzyme inhibition and molecular docking were used to verify the effective ingredients. **Results:** In vitro hypoglycemic activities of four extracts of SP (SP-10/SP-40/SP-60/SP-C) showed that SP-10 exhibited strong *α*-glucosidase (sucrase and maltase) inhibitory effects with IC_50_ of 67.81 μg/mL and 62.99 μg/mL, respectively. Carbohydrate tolerance assays demonstrated that SP-10 could significantly reduce the PBG levels of diabetic mice, with a significant hypoglycemic effect at a dosage of 20 mg/kg. A total of 26 constituents, including 11 caffeoylquinic acids (CQAs) and 15 flavonol glycosides, were tentatively identified by mainly analyzing secondary MS fragmentation. Moreover, three CQAs rich in SP-10, namely chlorogenic acid (CGA), neochlorogenic acid (NCGA), and cryptochlorogenic acid (CCGA), may be the main hypoglycemic substances, as evidenced by their inhibitory effects on sucrase and maltase. **Conclusions:** The *α*-glucosidase inhibitory effects of SP extract both in vitro and in vivo and its active ingredients were systematically studied for the first time. Results indicated that SP extract, rich in CQAs, had significant hypoglycemic activity, supporting the considerable potential of SP as hypoglycemic functional food or cost-effective therapeutic agents for diabetes treatment.

## 1. Introduction

Diabetes mellitus (DM), with its high incidence and difficulty to cure, has become the world’s most common chronic disease, imposing a substantial health and economic burden on patients and society [1]. According to the data from the International Diabetes Federation (IDF), the total number of diabetes patients is expected to rise to 853 million by 2050, and diabetes caused 3.4 million deaths in 2024 (IDF, https://diabetesatlas.org/) URL (accessed on 19 June 2025). Long-term hyperglycemia, especially the violent fluctuation of postprandial blood glucose (PBG), is the main cause of various organ injuries, cardiovascular and cerebrovascular disease, renal injury, neuropathy, and other diabetic complications [2,3]. Therefore, reducing PBG and maintaining blood glucose homeostasis have always been one of the important strategies for diabetes treatment. As a key enzyme for digestion and absorption of carbohydrates in the small intestine, *α*-glycosidase plays a central role in regulating PBG levels [4,5]. In fact, *α*-glycosidase inhibitors (AGIs) have become the first-choice drugs to control PBG, especially in Asian countries where rice and wheat are staple foods. Currently, the most commonly utilized AGIs in clinical practice include acarbose, miglitol, and voglibose. However, all these drugs are associated with side effects, such as abdominal cramps, diarrhea, flatulence, and vomiting, and are very expensive [3,6]. Therefore, the search for novel AGIs, especially those isolated from natural sources or plant-derived extracts, has attracted high attention from researchers aiming to develop hypoglycemic agents with enhanced efficacy and reduced toxicity [7,8].

*Silphium perfoliatum* L. (SP), also known as the cup plant, is a perennial member of Silphium genus in the family Asteraceae, native to North America [9]. Because of its nourishing properties and medicinal ability to treat liver, spleen, and digestive system diseases, it has been a traditional medicinal plant of Indigenous tribes [10,11,12]. In recent years, due to its excellent growth performance, rich nutritional value, and diverse biological activities, this plant has been widely planted and applied around the world [13]. Accordingly, more and more attention has been paid to its chemical composition and biological activity. Earlier phytochemical studies have demonstrated that the chemical composition of SP mainly included polysaccharides [14], volatile oils [15,16], flavonoids [17,18], and phenolic acids [19,20]. However, compared with most medicinal plants, the comprehensive analysis and structural elucidation of the chemical components of this plant are still seriously lagging. This not only greatly restricts the study of its traditional efficacy and its material basis but also hinders the discovery of new active ingredients and compounds. This also leads to the fact that the pharmacological activity research of SP is still in its infancy. Relevant studies have only preliminarily confirmed that SP extract has antibacterial activity, and its polysaccharide extract has displayed antioxidant and hypoglycemic activities in vitro [14,21]. In recent years, our research group obtained the active component rich in caffeoylquinic acid compounds (CQAs) from SP and confirmed that it possesses hepatoprotective effects on cholestatic mice by regulating enterohepatic circulation of bile [22], and could improve lipid accumulation in NAFLD mice by regulating the AMPK/FXR signaling pathway [23]. It is worth noting that SP is rich in polyphenolic substances, especially CQAs, which have been proven to have strong *α*-glucosidase inhibitory activity [24,25]. However, to date, there has been no study comprehensively evaluating the in vitro and in vivo hypoglycemic effects of SP by *α*-glucosidase inhibition and determining the chemical constituents responsible for reducing PBG in the SP active site using UPLC-Triple-TOF-MS/MS.

In this study, the inhibitory activities against *α*-glucosidase (including sucrase and maltase) of four different leaf extracts (SP-C, SP-10, SP-40, and SP-60) from SP were studied, and the PBG-lowering effects in diabetes mice of the most potent active extract SP-10 were also evaluated for the first time. Furthermore, the chemical compositions of SP-10 were characterized by UPLC-Triple-TOF-MS/MS, and the hypoglycemic activities of the main components in SP were further evaluated by sucrase and maltase inhibition assays and molecular docking.

## 2. Results

### 2.1. α-Glucosidase Inhibitory Effects of SP Extracts In Vitro

**Figure 1:**
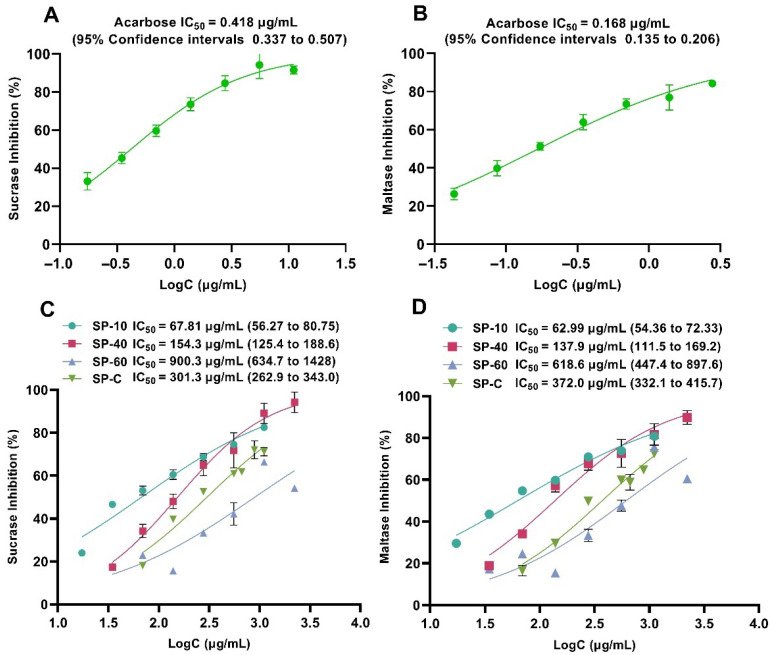
Inhibitory activities of acarbose and SP different extracts on sucrase and maltase in vitro. The IC_50_ values on sucrase (**A**) and maltase (**B**) of acarbose; the IC_50_ values on sucrase (**C**) and maltase (**D**) of SP-10/SP-40/SP-60 and SP-C.

We initially performed in vitro experiments to assess the inhibitory effects of SP extracts (SP-C, SP-10, SP-40, SP-60) on *α*-glucosidase (including sucrase and maltase) to screen the most potent inhibitory fraction. As shown in Figure 1, the IC_50_ values of acarbose for sucrase and maltase were 0.418 μg/mL (95% CI: 0.337 to 0.507) and 0.168 μg/mL (95% CI: 0.135 to 0.206), respectively. The IC_50_ values of SP-10, SP-40, SP-C, and SP-60 for sucrase inhibition were 67.81 μg/mL, 154.3 μg/mL, 301.3 μg/mL, and 900.3 μg/mL, respectively, which were 162, 369, 721, and 2154 times higher than that of acarbose (Figure 1A,C). For the maltase inhibition, the IC_50_ values were 62.99 μg/mL (SP-10), 137.9 μg/mL (SP-40), 372.0 μg/mL (SP-C), and 618.6 μg/mL (SP-60). Compared to acarbose, the IC_50_ values were approximately 375, 821, 2214, and 3682 times higher, respectively (Figure 1B,D). Based on the IC_50_ values, their inhibitory activities followed the order: SP-10 > SP-40 > SP-C > SP-60, with SP-10 exhibiting superior inhibitory effects against both sucrase and maltase compared to other extracts. Therefore, SP-10 was selected for subsequent in vivo evaluation to validate its potential in attenuating postprandial hyperglycemia.

### 2.2. PBG-Lowering Effects of SP-10 in Diabetic Mice

**Figure 2:**
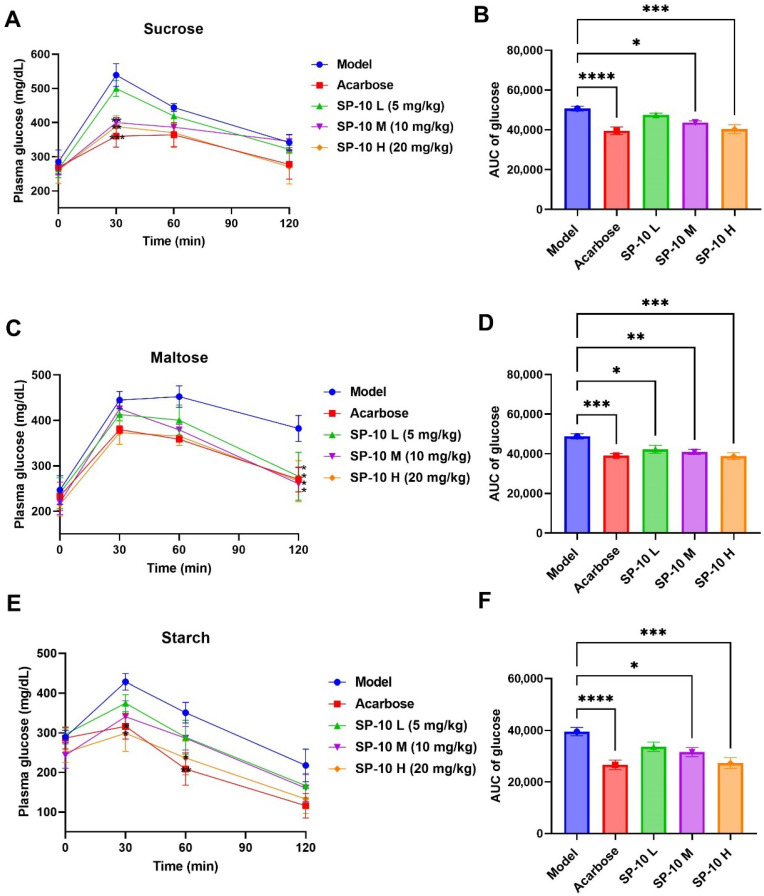
The effects of acarbose and SP-10 on reducing PBG in vivo. (**A**,**C**) and (**E**) curves showing PBG levels of diabetic mice after loading SP-10 or acarbose with sucrose (**A**), maltose (**C**), and starch (**E**) within 120 min. (**B**,**D**) and (**F**): incremental AUC_0–120 min_ of diabetic mice after administrating sucrose (**B**), maltose (**D**), and starch (**F**). Data were expressed as the mean ± SEM (*n* = 8; ****, *p* < 0.0001; ***, *p* < 0.001; **, *p* < 0.01; *, *p* < 0.05; compared with the diabetic model group).

The hypoglycemic effects of SP-10 in diabetic mice were further evaluated through carbohydrate tolerance tests, including starch, sucrose, and maltose. Continuous glucose monitoring was performed at 0, 30, 60, and 120 min following the oral administration of carbohydrate solutions (3 g/kg of body weight). The AUC for blood glucose levels was also calculated, as shown in Figure 2. After sucrose intake, the model group showed a peak PBG at 30 min and a gradual decline over the next 90 min; however, it still remained higher than pre-meal levels at 120 min. Compared to the model group, the positive control group and SP-10-treated groups (receiving 10 and 20 mg/kg doses) exhibited significant attenuation in PBG levels at 30 min. More importantly, both acarbose and SP-10 H groups successfully restored glycemic levels to preprandial values by 120 min (Figure 2A). Furthermore, the blood glucose AUC in diabetic mice treated with acarbose and SP-10 (at 10 and 20 mg/kg) was significantly lower than that in the model group (Figure 2B). The results demonstrated that dose-dependent SP-10 reduced PBG levels in diabetic mice when co-administered with sucrose. Specifically, the PBG-lowering effects of SP-10 at 20 mg/kg were comparable to those of acarbose.

Following maltose administration, PBG levels in the model group rapidly peaked at 30 min, maintained elevation until 60 min, then gradually declined but remained relatively high at 120 min. As shown in Figure 2C, all three SP-10 treatment groups and the acarbose group had lower PBG levels at 30 and 60 min, although these differences were not statistically significant. However, at 120 min, all treatment groups exhibited statistically significant reductions in PBG levels compared to the model group. Moreover, AUC values in SP-10 treatment groups at various concentrations were significantly decreased compared to those in the model group (Figure 2D). It is worth emphasizing that the hypoglycemic effects of SP-10 administered at 20 mg/kg were equivalent to those of acarbose.

In the starch tolerance test, the PBG of the model group reached peak values at 30 min and then decreased in a gradual manner. Compared to the model group, both the SP-10 H group and the acarbose group demonstrated significantly reduced PBG levels at all measured time points, with similar reduction effects observed between the two groups (Figure 2E). In addition, the AUC levels of acarbose, SP-10 M, and SP-10 H groups were markedly decreased compared to the model group (Figure 2F). These findings demonstrated that SP-10 (20 mg/kg) and acarbose (6 mg/kg) showed comparable efficacy in reducing the PBG level in diabetic mice following starch ingestion. Integrated analysis of carbohydrate tolerance tests revealed that SP-10 could significantly reduce PBG levels in diabetic mice, with a clear dose–response relationship. Notably, SP-10 administration at 20 mg/kg showed significant antihyperglycemic efficacy, indicating its therapeutic potential for diabetes.

### 2.3. Chemical Profiling of SP-10

**Figure 3:**
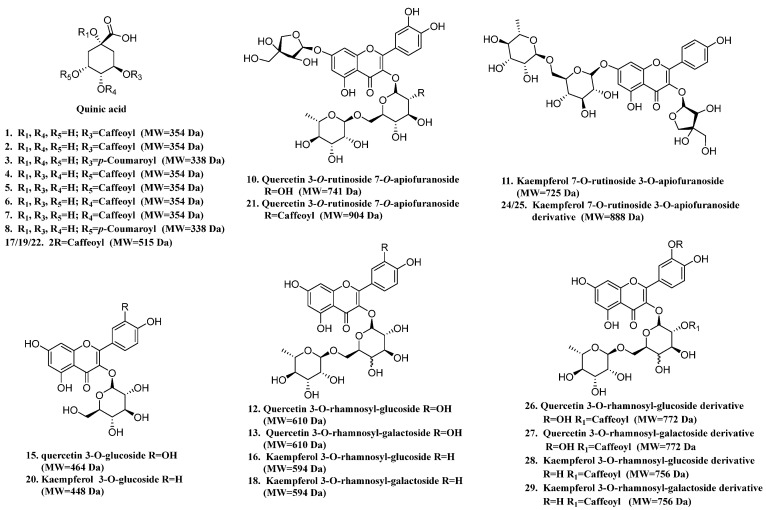
Structural skeletons of phenolic components in SP-10.

Based on the potent hypoglycemic effect of SP-10, comprehensive phytochemical analysis was conducted using UPLC-Triple-TOF-MS/MS. A total of 26 compounds including 11 CQAs and 15 flavonol glycosides (Table 1, Figure 3) were tentatively identified by mainly analyzing secondary MS fragmentation as well as comparison with references.

Compounds **1**, **2**, **4**, **5**, **6**, and **7** all gave molecular ions [M-H]^-^ at *m*/*z* 353, and produced base peaks [M-H-162]^-^ at *m*/*z* 191 and fragment ions [M-H-174-44]^-^ at *m*/*z* 135; however, the peak intensity at *m*/*z* 135 from compounds 4 and 5 was relatively weak. Then, compounds **1**, **2**, **4**, **5**, **6**, and **7** were identified as 3-monocaffeoylquinic acid I, 3-monocaffeoylquinic acid II, 5-monocaffeoylquinic acid I, 5-monocaffeoylquinic acid II, 4-monocaffeoylquinic acid I, and 4-monocaffeoylquinic acid II based on the literature [26,27,28]. Compounds **3** and **8**, with molecular ions [M-H]^-^ at *m*/*z* 337, were identified as 3-p-coumaroylquinic acid and 5-p-coumaroylquinic acid due to the presence of characteristic fragment ions [M-H-146]^-^ at *m*/*z* 191 and [M-H-174]^-^ at *m*/*z* 119 [26,28]. In addition to monocaffeoylquinic acids, three dicaffeoylquinic acids have also been identified from this plant. Compounds **17**, **19**, and **22**, all with molecular ions [M-H]^-^ at *m*/*z* 515, were determined as dicaffeoylquinic acids according to the presence of characteristic fragment ions [M-H-162]^-^ at *m*/*z* 353, [M-H-162-162]^-^ at *m*/*z* 191, and [M-H-162-174-44]^-^ at *m*/*z* 135 [26,28].

Two aglycones of flavone glycosides, including quercetin (aglycone of compounds **10**, **12**, **13**, **15**, **21**, **26**, and **27**) and kaempferol (aglycone of compounds **11**, **16**, **18**, **20**, **24**, **25**, **28**, and **29**) were detected from this plant. Generally, the main sugar structure types of flavonoid glycosides are pentose (xylose, arabinose), 6-deoxyhexose (xylose, furanose), and hexose (galactose, glucose).

Compounds **10** and **11**, with [M-H]^-^ at *m*/*z* 741 and *m*/*z* 725, were illustrated as quercetin 3-*O*-rutinoside-7-*O*-apiofuranoside and kaempferol 7-*O*-rutinoside-3-*O*-apiofuranoside [29,30,31]. Both compounds produced characteristic aglycone ions [M-H-132-308]^-^ at *m*/*z* 301 and *m*/*z* 285, and free radical aglycone ions at *m*/*z* 300 and at *m*/*z* 284, respectively, by sequentially losing arabinose (-132 u) and rutinose (-308 u). Similarly, compounds **21** and **24**/**25** were determined as quercetin 3-*O*-rutinoside-7-*O*-apiofuranoside derivative and kaempferol 7-*O*-rutinoside-3-*O*-apiofuranoside derivative due to the generation of characteristic aglycone ions at *m*/*z* 301 and *m*/*z* 285, and fragment ions [M-H-132]^-^ at *m*/*z* 771 and *m*/*z* 755, respectively [29,30,31]. Compounds **15** and **20**, with [M-H]^-^ at *m*/*z* 463 and *m*/*z* 447, yielded signature aglycone ions [M-H-162]^-^ at *m*/*z* 301 and *m*/*z* 285 via glucose loss (-162 u), matching reference spectra of quercetin 3-*O*-glucoside and kaempferol 3-*O*-glucoside [29,30]. Peaks **12** and **13**, isobaric [M-H]^-^ at *m*/*z* 609, produced the same characteristic aglycone ion [M-H-162]^-^ at *m*/*z* 301 and free radical aglycone ion at *m*/*z* 300, and were distinguished as quercetin 3-*O*-rhamnosyl-glucoside and quercetin 3-*O*-rhamnosyl-galactoside [32]. Peaks **16** and **18**, with isobaric [M-H]^-^ at *m*/*z* 593, produced the same aglycone ion [M-H-162]^-^ at *m*/*z* 285 and free radical aglycone ion at *m*/*z* 284, and were characterized as kaempferol 3-*O*-rhamnosyl-glucoside and kaempferol 3-*O*-rhamnosyl-galactoside [32]. Compounds **26** and **27**, with [M-H]^-^ at *m*/*z* 771, produced characteristic aglycone ion [M-H-162-308]^-^ at *m*/*z* 301, as well as free radical aglycone ions at *m*/*z* 300, by sequentially losing caffeoyl (-132 u) and rutinose (-308 u); thus, these were deduced as caffeoyl-quercetin 3-*O*-rhamnosyl-glucoside [32]. Similarly, Compounds **28** and **29** were inferred as caffeoyl-kaempferol 3-*O*-rhamnosyl-glucoside [31].

### 2.4. The Main Compounds of SP-10 Inhibit α-Glucosidase In Vitro

**Figure 4:**
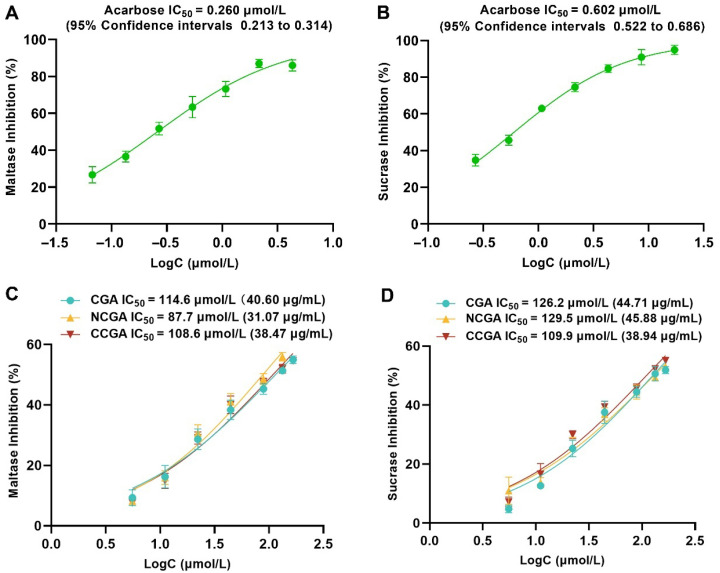
The IC_50_ values of acarbose and the three CQAs in SP-10 on maltase and sucrase in vitro. The IC_50_ values on maltase (**A**) and sucrase (**B**) of acarbose; the IC_50_ values on maltase (**C**) and sucrase (**D**) of CGA, NCGA, and CCGA.

The *α*-glucosidase inhibitory activities of the three predominant CQAs (CGA, NCGA, CCGA) in SP-10 were evaluated in vitro. As shown in Figure 4, the IC_50_ values against maltase of CGA, NCGA, and CCGA were 114.6 μmol/L (95% CI: 101.4–131.4), 87.7 μmol/L (95% CI: 76.8–102.0), and 108.6 μmol/L (95% CI: 95.3–125.8), respectively, with NCGA showing superior inhibition. As for sucrase, the IC_50_ values were CGA 126.2 μmol/L (95% CI: 109.1–149.5), NCGA 129.5 μmol/L (95% CI: 114.6–148.7), and CCGA 109.9 μmol/L (95% CI: 96.4–127.3). The acarbose IC_50_ values for maltase and sucrase were 0.260 μmol/L (95% CI: 0.213–0.314) and 0.602 μmol/L (95% CI: 0.522–0.686), respectively. Relative to acarbose, CGA, NCGA, and CCGA exhibited higher IC_50_ against maltase (441-, 337-, and 418-fold) and sucrase (210-, 215-, and 182-fold), respectively. The results demonstrated that under in vitro conditions, all three CQAs showed good inhibitory activities against glucosidase, especially against sucrase, which was stronger than maltase.

Meanwhile, compared to SP-10 extract, the three CQAs showed stronger *α*-glucosidase inhibitory activity, as evidenced by their obviously lower IC_50_ values (Figure 1, Figure 2, Figure 3 and Figure 4). In comparison, these compounds exhibited 1.4–2.0-fold greater inhibitory potency than SP-10, thus confirming their role as key hypoglycemic constituents underlying SP-10’s glucose-regulating activity.

### 2.5. Molecular Docking Results

**Figure 5:**
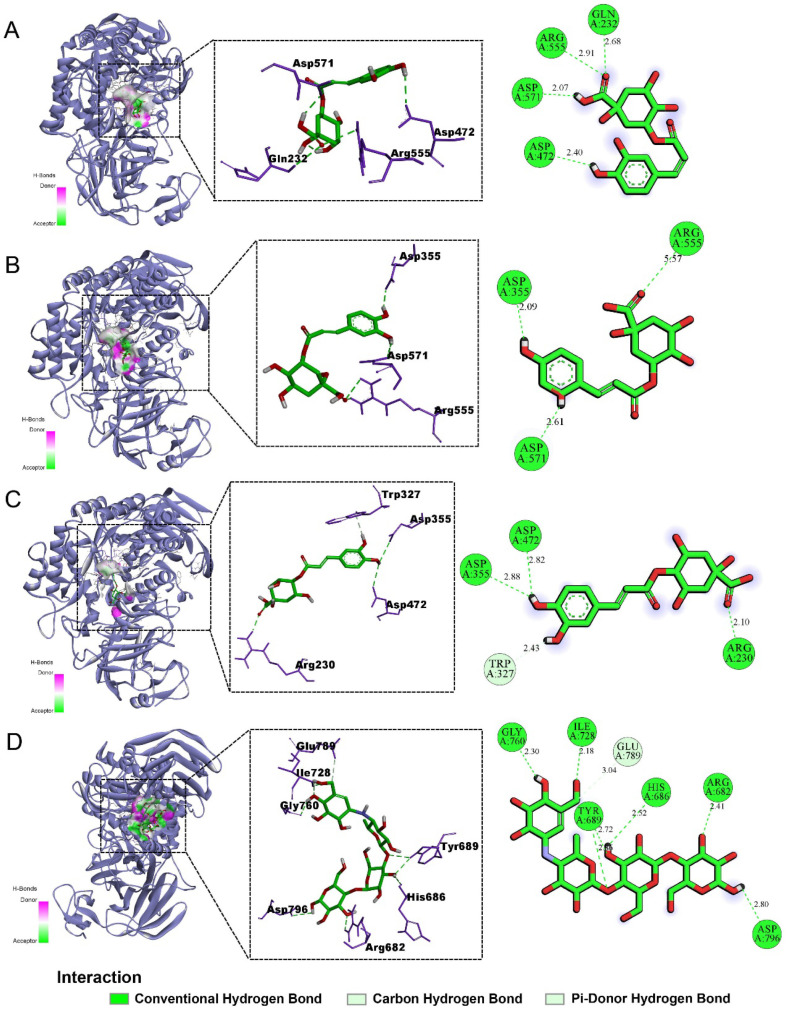
The molecular docking study of the three CQAs and acarbose bound to sucrase. (**A**) CGA; (**B**) NCGA; (**C**) CCGA; (**D**) acarbose. From left to right, there are overall, partial, and 2D images in sequence. Cartoons represent proteins, while sticks represent small molecules. Green dashed lines represent hydrogen bonding interactions.

**Figure 6:**
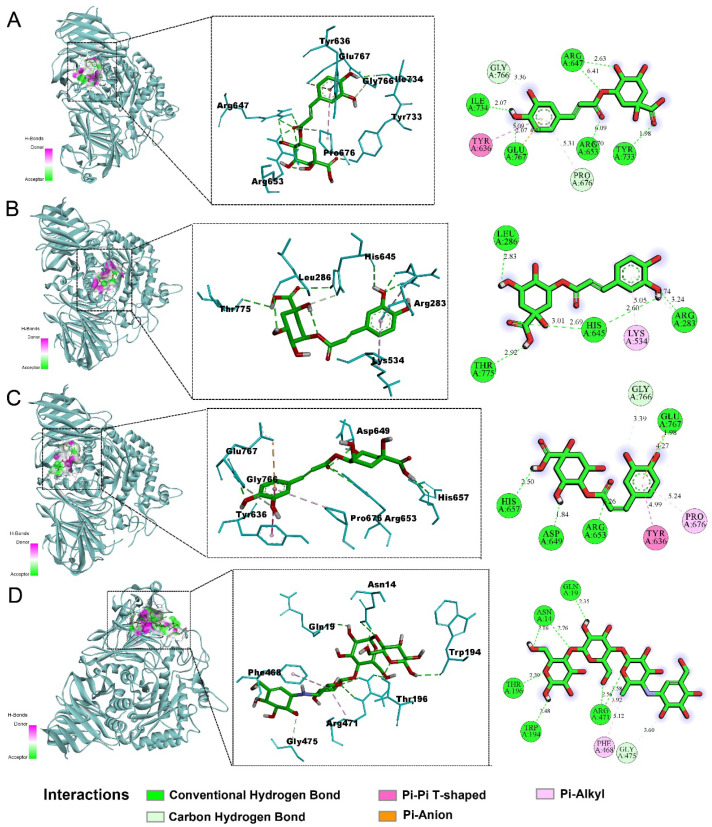
The molecular docking study of the three CQAs and acarbose bound to maltase. (**A**) CGA; (**B**) NCGA; (**C**) CCGA; (**D**) acarbose. From left to right, there are overall, partial, and 2D images in sequence. Cartoons represent proteins, while sticks represent small molecules. Green, pink, and orange dashed lines represent hydrogen bonding and hydrophobic and electrostatic interactions, respectively.

Molecular docking simulations were performed to further elucidate the inhibitory mechanisms of CGA, NCGA, and CCGA against sucrase and maltase. The simulation results revealed that all three CQAs formed stable binding conformations within catalytic pockets of both enzymes through hydrogen bonding, electrostatic interactions, and hydrophobic contacts (Figure 5 and Figure 6). Hydrogen-bond network density correlated directly with inhibitory potency, where increased hydrogen bonding enhanced binding affinity and enzyme inhibition [33]. Notably, sucrase inhibition occurred exclusively through hydrogen bonding, indicating superior inhibiting effects. Generally, lower binding energy is associated with more stable conformations and stronger affinity. The binding energy results (Table 2 and Table 3) showed relatively low docking scores between the three CQAs and both sucrase and maltase; particularly, all of the compounds displayed comparatively lower binding energies for sucrase than acarbose, indicating a more stable structure. These findings were consistent with the results of in vitro experiments. In summary, the molecular docking results confirmed that CGA, NCGA, and CCGA possessed strong binding abilities with maltase, especially sucrase, thereby inhibiting *α*-glucosidase activity and contributing to the reduction of PBG levels.

## 3. Discussion

Plant-derived *α*-glucosidase inhibitors exhibit diverse structural types; however, those with potency comparable to the first-line medication acarbose are still yet to be discovered. Therefore, identifying medicinal plants possessing potent *α*-glucosidase inhibitory activity and isolating their active components or monomeric compounds are major focuses of current research in this field [8,33,34]. In this study, the glycosidase inhibitory activities and hypoglycemic effects of SP-10 derived from SP were systematically evaluated for the first time through integrated in vitro and in vivo assays, and the structure of this component was further characterized using UPLC-Triple-TOF-MS/MS. The results demonstrated that SP-10, obtained via in vitro screening, exhibited potent *α*-glucosidase inhibitory activity, with a dosage of 20 mg/kg showing significant antihyperglycemic effects in vivo. It is noteworthy that SP-10 is rich in CQAs, exhibiting both structural diversity and high abundance. Among the 26 identified phenolic compounds, 15 were flavonol glycosides, while the remaining 11 were CQAs. In a previously published article, quantitative analysis results showed that the contents of NCGA, CGA, and CCGA in SP-10 were 22.1%, 19.0%, and 8.9% [23]. Notably, NCGA, CGA, and CCGA were particularly abundant, suggesting that these compounds may be the main active substances in its hypoglycemic effect. This study provides the first evidence that SP possesses great potential for reducing PBG levels, and that its extract SP-10 serves as a potential active component for glycosidase inhibitors. Moreover, in our previous experiments, no mortality or adverse effects were observed in mice administered the maximum dose of 400 mg/kg over a 14-day period [22,23]. As a traditional medicinal plant, SP has strong adaptability, high biomass yield, and diverse biological activities, making it a highly promising candidate plant for diabetes treatment [13,35,36].

Because an increasing number of plant extracts or compounds have been discovered to possess *α*-glucosidase inhibitory activity, the development of safe and efficient hypoglycemic drugs from plant sources may be realized [37,38]. However, it is disheartening that plant-derived glycosidase inhibitors with in vivo glucose-lowering effects comparable to acarbose are still extremely limited. Notably, while numerous studies confirm superior in vitro glycosidase inhibition by certain plant extracts compared to acarbose, their in vivo performance often differs significantly [39,40]. This discrepancy may be related not only to the absorption and metabolism of the compounds in vivo but also to the source of glycosidases used in the screening systems. Currently, the in vivo dosage of plant-based glycosidase inhibitors mostly reaches several hundred milligrams per kilogram (mg/kg of body weight), yet it is still challenging to achieve the hypoglycemic efficacy of acarbose [41,42,43,44]. In contrast, SP-10 in this study, despite showing lower in vitro glycosidase inhibitory activity than the positive control, achieved significant glucose reduction at a dose of 10 mg/kg in vivo. Furthermore, at 20 mg/kg, its hypoglycemic effect matched acarbose. Due to its strong hypoglycemic activity in vivo, it has been confirmed that SP has considerable potential for developing glycosidase inhibitors or hypoglycemic agents. Based on these findings, further investigation into its chemical constituents is warranted to elucidate the structural types of compounds and identify potent inhibitors.

Current research on the chemical constituents of SP remains relatively limited, particularly lacking systematic investigations into the material basis responsible for its traditional effects, such as hepatoprotective and hypoglycemic activities. In this study, compounds from SP were preliminary separated and enriched using macroporous resin. The resulting four fractions were screened for *α*-glucosidase inhibitory activity. Subsequently, utilizing UPLC-Triple-TOF-MS/MS analysis, 26 phenolic compounds were identified from the most active fraction, SP-10, including 15 flavonol glycosides and 11 CQAs. Both classes of compounds have been reported to exhibit glycosidase inhibitory activity [45,46,47,48]. Among them, CGA, a representative compound within CQAs, has garnered widespread attention owing to its diverse bioactivities, and its glycosidase inhibitory effect has been confirmed by multiple studies [24,25,49]. However, the inhibitory activities of its structural analogues and isomers have not yet received commensurate research attention. SP-10 contains not only CGA but also NCGA and CCGA as major constituents, alongside eight structurally distinct CQAs (including dicaffeoylquinic acids, diCQAs). Both in vitro activity studies and molecular docking confirmed strong *α*-glucosidase inhibitory activities by NCGA, CGA, and CCGA, suggesting their role as primary active components in SP-10’s hypoglycemic effect. While the remaining eight CQAs occur at lower abundance in SP-10, future research should also focus on their in vitro and in vivo activities. LC-MS analysis and in vitro studies indicated that the CQAs in SP were not only abundant but also diverse in structural types, making them representative compounds of this plant. Based on our previous research, these components play important roles in regulating glucose and lipid metabolism, as well as treating metabolic diseases.

Additionally, the observed discrepancy between the in vitro and in vivo glucosidase inhibitory activity of SP-10 should also be considered. In vitro assays revealed that the IC_50_ values of SP-10-inhibiting sucrase and maltase were 162-fold and 375-fold higher, respectively, than those of acarbose, indicating its weaker inhibitory activity. However, interestingly, in vivo studies demonstrated that SP-10 (20 mg/kg) achieved PBG-lowering efficacy comparable to that of acarbose (6 mg/kg) in diabetic mice. Does the difference in activities between in vitro and in vivo suggest that the hypoglycemic mechanism of SP-10 extends beyond *α*-glucosidase inhibition and may involve other pathways? In our previous research, we confirmed that SP-10 exerts a protective effect on cholestatic liver injury by regulating the enterohepatic circulation of bile acids [22]. Additionally, another study reported that SP-10 ameliorates lipid accumulation in NAFLD mice by modulating the AMPK/FXR signaling pathway [23]. Both lipid metabolism and bile acid metabolism are potentially associated with glucose and lipid metabolism. Specifically, does this component participate in regulating glucose metabolism via modulation of hepatic and biliary functions, or is its action on glycosidase not limited to inhibition alone? These potential mechanisms are all worthy of further investigation. Thus, this study preliminarily confirmed the hypoglycemic efficacy of SP-10 through in vitro screening and in vivo activity studies and initially clarified the structural types of its compounds. These findings provide foundational data for expanding glycosidase inhibitor sources and advancing SP development.

## 4. Materials and Methods

### 4.1. Materials and Reagents

Acarbose (≥98% purity, Lot# DSTDA003501), sucrose, and maltose were procured from Solaibio Biotechnology Co., Ltd. (Beijing, China). Soluble starch was sourced from Tianjun Biotechnology Co., Ltd. (Guangzhou, China). The glucose assay kit was acquired from Shanghai Rongsheng Biopharmaceutical Co., Ltd. (Shanghai, China). Reference standards of chlorogenic acid (CGA), cryptochlorogenic acid (CCGA), and neochlorogenic acid (NCGA) were previously isolated and characterized in our laboratory from *S. perfoliatum*. Ultrapure water used in this study was generated by a Milli-Q Biocel water purification system (Millipore, Bedford, MA, USA).

### 4.2. Preparation of Plant Extracts

SP leaves were collected in July from Huzhu County, Qinghai Province, China. The taxonomic authentication of *S. perfoliatum* was performed by Dr. Xiaofeng Chi, with the corresponding voucher specimen (Accession No. chi2023559) being permanently archived in the Qinghai-Tibetan Plateau Museum of Biology. The extraction processes were carried out in accordance with our previously reported method with slight modifications [22]. Dry leaves (5.0 kg) were extracted three times with 70% ethanol (1:10, *w*/*v*; 50 L per cycle) at 70 °C, and the combined extracts were concentrated under reduced pressure using a rotary evaporator (IKA RV10, IKA, Staufen, Germany) to obtain crude extract SP-C. Then, SP-C was purified and enriched on a D-101 macroporous resin column. After removing sugar with pure water, SP-C was eluted with ethanol–water gradients (10:90, 40:60, and 60:40 *v*/*v*). Corresponding eluates were concentrated and freeze-dried to receive fractions SP-10 (10% ethanol), SP-40 (40% ethanol), and SP-60 (60% ethanol) for further research.

### 4.3. Animals

Male Sprague Dawley rats (6-week-old, 180–220 g) and Kunming mice (6-week-old, 18–22 g) were obtained from Sibeifu Co., Ltd. (Beijing, China; Certification No. SCXK-[Jing]-2019-0010). All animals were maintained in SPF-class facilities under the following temperature conditions: 23 ± 1 °C; relative humidity: 50 ± 5%, with 12-h light/dark cyclicity, and standard rodent chow and purified water were provided. SD rats were used to extract the *α*-glucosidase from the intestinal mucosa, while KM mice were employed to conduct the carbohydrate tolerance tests. Before the experiments, all animals underwent one week of adaptive feeding. All experimental protocols were approved by the Animal Ethics Committee of the Northwest Institute of Plateau Biology, CAS.

### 4.4. Preparation and Inhibition Assay of α-Glucosidase

The *α*-Glucosidase preparation and inhibitory activity evaluation followed established laboratory protocols [41]. After overnight fasting, rats were humanely euthanized according to approved ethical guidelines. Small intestinal segments were immediately excised and placed on ice to preserve enzymatic activity. Following longitudinal dissection, the luminal surface was rinsed three times with pre-chilled PBS (0.1 M, pH 7.4), and mucosa was scraped using sterile slides. The mucosal tissue was subsequently dissolved in cold PBS (1:5 *m*/*v*), centrifuged (8000 rpm, 4 °C, 4 min), and the supernatant was snap-frozen in liquid nitrogen for storage at −80 °C.

The *α*-Glucosidase inhibition was assayed by incubating 50 μL of maltose (1 mmol/L) or sucrose (50 mmol/L) with 50 μL of test samples (SP-C, SP-10, SP-40, SP-60, acarbose, or individual compounds) in 48-well plates. After shaking, 50 μL of maltase (11.32 U/mL) or sucrase (15.26 U/mL) was added and incubated at 37 °C for 20 min with oscillation. Reactions were terminated by heating at 95 °C for 15 min. Glucose production was quantified using a glucose assay kit, and IC_50_ values were calculated with GraphPad Prism 9.5.1.

### 4.5. PBG Detection in Diabetic Mice

PBG detection in diabetic mice adhered to the protocol previously established by our group [41]. After 12-h fasting, hyperglycemia was induced by tail vein injection of alloxan at a dose of 60 mg/kg. Fasting blood glucose was measured 72 h post-induction. Mice with blood glucose levels of 180–360 mg/dL were considered diabetic and randomized into five groups (*n* = 8/group): Model (sucrose 3 g/kg); Acarbose (sucrose 3 g/kg + acarbose 6 mg/kg); SP-10 L (sucrose 3 g/kg + SP-10 5 mg/kg); SP-10 M (sucrose 3 g/kg + SP-10 10 mg/kg) and SP-10 H (sucrose 3 g/kg + SP-10 20 mg/kg). Blood glucose was measured pre-gavage and at 30, 60, and 120 min post-gavage. Data was recorded and analyzed for PBG and AUC using Graphpad Prism 9.5.1. Maltose and starch experiments used identical designs.

### 4.6. UPLC-Triple-TOF-MS/MS Analysis

Phytochemical analysis was conducted using a UPLC-Triple-TOF-MS/MS system equipped with an electrospray ionization (ESI) source (Acquity UPLC, Waters; Triple TOF 5600+, AB SCIEX, Marlborough, MA, USA). SP-10 extract was dissolved in HPLC-grade methanol, ultrasonicated (10 min), and centrifuged (10,000 rpm, 30 min), and the supernatant was analyzed. Separation was performed on a BEH-C18 column (150 mm × 2.1 mm, 1.7 μm; Waters, Milford, MA, USA) with the column temperature set at 40 °C. The mobile phase consisted of 0.1% (*v*/*v*) formic acid in ultrapure water (A) and acetonitrile (B), with the following gradient elution: 0–10 min, 98–80% A (*v*/*v*); 10–25 min, 80–5% A (*v*/*v*). The flow rate was set at 0.3 mL/min with a 3 μL injection volume.

Mass spectrometric detection employed the following parameters: ion source temperatures, negative mode 550 °C, and positive mode 600 °C; ion source voltages, negative mode −4500 V, and positive mode 5500 V; atomized gas, 50 psi; curtain gas, 35 psi. In primary scan mode, the focusing voltage was 10 V and the de-clustering voltage was 100 V. For tandem MS analysis, mass spectrum data were acquired in TOF-MS/MS-IDA mode with CID energies of ramped from −60 V to −20 V in 20 eV increments. Instrument calibration was performed preceding analytical runs, with mass axis alignment achieved through continuous infusion of calibrant solution (CDS Pump, AB SCIEX, Marlborough, MA, USA; 500 μL/min) to maintain mass accuracy below 2 ppm. The mass range for analysis was set from 100 to 1500 *m*/*z*. Dual-polarity detection was implemented with alternating positive/negative ionization modes for all samples.

### 4.7. Molecular Docking

The crystal structures of sucrase and maltase were retrieved from the Protein Data Bank (PDB). The three-dimensional structures of CGA, CCGA, and NCGA were acquired from the PubChem chemical repository (https://pubchem.ncbi.nlm.nih.gov/) URL (accessed on 4 November 2024). Protein structures were pre-processed in PyMOL 3.0 to add hydrogen atoms, remove water molecules, and define the grid box. Structure-based virtual screening was conducted via AutoDock Vina (v1.1.2) with empirically validated parameters. Top-scoring conformations were further visualized and analyzed in Discovery Studio 2019 Client software.

### 4.8. Statistical Data Processing

Experimental data were expressed as mean ± standard error of the mean (SEM), derived from at least three independent experimental replicates. Statistical analyses were performed using GraphPad Prism. One-way analysis of variance (ANOVA) and subsequent Bonferroni post hoc test were conducted to evaluate significant differences among groups. The values of *p* < 0.05 were considered statistically significant. Nonlinear regression was employed to determine the half-maximal inhibitory concentration (IC_50_) for enzyme activity inhibition.

## 5. Conclusions

In summary, we demonstrated that SP-10 exhibited strong *α*-glucosidase inhibitory activities (IC_50_: 67.81 μg/mL for sucrase, 62.99 μg/mL for maltase) and significantly reduced PBG levels in diabetic mice. Impressively, it had a strong hypoglycemic effect at an in vivo dose of 20 mg/kg, indicating high potency at a relatively low dose. Furthermore, LC-MS/MS analysis tentatively identified 26 phenolic compounds in SP-10, including 11 CQAs and 15 flavonol glycosides, and enzyme inhibition assays combined with molecular docking further indicated that CGA, NCGA, and CCGA, which were abundant in SP-10, may be the main hypoglycemic substances. These findings support the potential of *S. perfoliatum* as a hypoglycemic functional food or a cost-effective therapeutic agent for diabetes treatment.

## Figures and Tables

**Table 1:** Identification of phenolic components in the SP-10 by UPLC-Triple-TOF-MS/MS.

Peak no.	RT (min)	[M-H]^-^	MS Fragments (*m*/*z*)	Proposed Compounds	Molecular Formula	References
1	6.65	353.0873	707.1815 (2M-H), 191.0550 (M-162), 135.0448 (179-44)	3-monocaffeoylquinic acid I	C_16_H_18_O_9_	[26,27,28]
2	7.27	353.0878	707.1818 (2M-H), 191.0546 (M-162), 135.0450 (179-44)	3-monocaffeoylquinic acid II	C_16_H_18_O_9_	[26,27,28]
3	9.31	337.0926	191.0541 (M-146), 163.0385 (M-174), 119.0498 (M-174)	3-*p*-coumaroylquinic acid	C_16_H_18_O_8_	[26,27,28]
4	9.46	353.0876	707.1823 (2M-H), 191.0555 (M-162)	5-monocaffeoylquinic acid I	C_16_H_18_O_9_	[26,27,28]
5	9.88	353.0878	707.1824 (2M-H), 191.0557 (M-162)	5-monocaffeoylquinic acid II	C_16_H_18_O_9_	[26,27,28]
6	10.19	353.0881	707.1823 (2M-H), 191.0554 (M-162), 135.0451 (179-44)	4-monocaffeoylquinic acid I	C_16_H_18_O_9_	[26,27,28]
7	10.55	353.0883	707.1823 (2M-H), 191.0557 (M-162), 135.0456 (179-44)	4-monocaffeoylquinic acid II	C_16_H_18_O_9_	[26,27,28]
8	12.81	337.0927	191.0559 (M-146), 163.0385 (M-174), 119.0507 (M-174)	5-*p*-coumaroylquinic acid	C_16_H_18_O_8_	[26,27,28]
9	12.02	337.0924		unknown	-	
10	15.20	741.1953	609.1510 (M-132), 301.0359 (M-132-162-146), 300.0277, 299.0203	quercetin 3-*O*-rutinoside 7-*O*-apiofuranoside	C_32_H_38_O_20_	[29,30,31]
11	16.58	725.2001	593.1544 (M-132), 416.0757, 285.0395 (M-132-162-146), 284.0317, 283.0236	kaempferol 7-*O*-rutinoside 3-*O*-apiofuranoside	C_32_H_38_O_19_	[29,30,31]
12	17.20	609.1503	301.0350 (M-308), 300.0270	quercetin 3-*O*-rhamnosyl-glucoside	C_27_H_30_O_16_	[32]
13	17.58	609.1494	301.0344 (M-308), 300.0266, 271.0237, 151.0030	quercetin 3-*O*-rhamnosyl-galactoside	C_27_H_30_O_16_	[32]
14	17.83	401.1822	221.1174, 177.1271	unknown	-	
15	18.25	463.0895	301.0354 (M-162), 271.0235, 255.0290, 243.0285, 151.0028	quercetin 3-*O*-glucoside	C_21_H_20_O_12_	[29]
16	18.90	593.1549	285.0397 (M-308), 284.0321, 255.0295, 227.0346	kaempferol 3-*O*-rhamnosyl-glucoside	C_27_H_30_O_15_	[32]
17	19.60	515.1243	353.0891 (M-162), 191.0558 (M-162-162), 173.0455, 135.0455 (179-44)	dicaffeoylquinic acid I	C_25_H_24_O_12_	[26,27,28]
18	19.76	593.1556	285.0400 (M-308), 284.0322, 255.0295	kaempferol 3-*O*-rhamnosyl-galactoside	C_27_H_30_O_15_	[32]
19	20.26	515.1185	353.0882 (M-162), 191.0560 (M-162-162), 179.0346, 135.0451 (179-44)	dicaffeoylquinic acid II	C_25_H_24_O_12_	[26,27,28]
20	20.56	447.0919	285.0383 (M-162), 284.0309, 255.0278, 227.0329	kaempferol 3-*O*-glucoside	C_21_H_20_O_11_	[30]
21	21.26	903.2303	771.1850 (M-132), 609.1495 (M-132-162), 433.0785, 301.0344	quercetin 3-*O*-rutinoside 7-*O*-apiofuranoside derivative	C_41_H_44_O_23_	[29,30,31]
22	21.65	515.1188	353.0886, 191.0549, 179.0340, 173.0445, 135.0445	dicaffeoylquinic acid III	C_25_H_24_O_12_	[26,27,28]
23	22.16	691.2615	335.1244, 317., 273.1241	unknown	-	
24	22.40	887.2357	755.1897 (M-132), 593.1544, 284.0313, 161.0232	kaempferol 7-*O*-rutinoside 3-*O*-apiofuranoside derivative I	C_41_H_44_O_22_	[29,30,31]
25	22.72	887.2371	755.1904 (M-132), 469.1354, 417.0828, 285.0396, 284.0320, 161.0237	kaempferol 7-*O*-rutinoside 3-*O*-apiofuranoside derivative II	C_41_H_44_O_22_	[29,30,31]
26	23.84	771.1885	609.1495 (M-162), 301.0340 (M-162-308), 300.0257, 178.9972	quercetin 3-*O*-rhamnosyl-glucoside derivative I	C_33_H_36_O_19_	[32]
27	24.14	771.1860	609.1507 (M-162), 301.0340 (M-162-308), 300.0264, 151.0026	quercetin 3-*O*-rhamnosyl-galactoside derivative II	C_33_H_36_O_19_	[32]
28	25.60	755.1908	593.1551 (M-162), 469.1362, 285.0397 (M-162-308)	kaempferol 3-*O*-rhamnosyl-glucoside derivative	C_33_H_36_O_18_	[31]
29	25.97	755.1888	593.1536 (M-162), 469.1350, 285.0388 (M-162-308)	kaempferol 3-*O*-rhamnosyl-galactoside derivative	C_33_H_36_O_18_	[31]

**Table 2:** The results of the three CQAs in SP-10 and acarbose bound to sucrase (3LPP).

Compounds	Affinity (Kcal/mol)	pKi (μmol/L)	H-Bonds	Hydrophobic	Electrostatic
Chlorogenic acid	−8.13 ± 0.34	1.24 ± 0.74	4	0	0
Neochlorogenic acid	−8.03 ± 0.21	1.35 ± 0.47	3	0	0
Cryptochlorogenic acid	−7.73 ± 0.15	2.20 ± 0.54	4	0	0
Acarbose	−7.30 ± 0.37	5.09 ± 2.97	8	0	0

**Table 3:** The results of the three CQAs in SP-10 and acarbose bound to maltase (2QMJ).

Compounds	Affinity (Kcal/mol)	pKi (μmol/L)	H-Bonds	Hydrophobic	Electrostatic
Chlorogenic acid	−8.43 ± 0.38	0.81 ± 0.69	7	2	1
Neochlorogenic acid	−8.10 ± 0.20	1.18 ± 0.40	5	2	0
Cryptochlorogenic acid	−8.53 ± 0.21	0.57 ± 0.21	5	2	1
Acarbose	−8.30 ± 0.36	0.91 ± 0.47	8	2	0

## Data Availability

The data presented in this study are available on request from the corresponding author. The data are not publicly available due to privacy.

## Abbreviations

The following abbreviations are used in this manuscript:

SP	*Silphium perfoliatum* L.
SP-C	SP crude extract
DM	Diabetes mellitus
AGIs	Alpha-glucosidase inhibitors
CI	Confidence intervals
AUC	Area under the curve
PBG	Postprandial blood glucose
CQAs	Caffeoylquinic acid compounds
CGA	Chlorogenic acid
CCGA	Cryptochlorogenic acid
NCGA	Neochlorogenic acid
